# MAP Kinase Phosphatase 3 (MKP3) Preserves Norepinephrine Transporter Activity by Modulating ERK1/2 Kinase-Mediated Gene Expression

**DOI:** 10.3389/fncel.2017.00253

**Published:** 2017-08-22

**Authors:** Ole V. Mortensen, Mads B. Larsen, Susan G. Amara

**Affiliations:** ^1^Department of Pharmacology and Physiology, Drexel University College of Medicine Philadelphia, PA, United States; ^2^Department of Cell Biology and Physiology, University of Pittsburgh School of Medicine Pittsburgh, PA, United States; ^3^National Institute of Mental Health Bethesda, MD, United States

**Keywords:** caveolin, dual-specificity phosphoprotein phosphatase, extracellular-signal-regulated kinase (ERK), mitogen-activated protein kinase (MAPK), monoamine transporter, norepinephrine, protein kinase C (PKC)

## Abstract

The norepinephrine transporter (NET) mediates the clearance of norepinephrine (NE) from the extracellular space and is a target of therapeutic antidepressants and psychostimulants. Previously we identified a MAP kinase phosphatase 3 (MKP3), as an important modulator of protein kinase C (PKC) mediated internalization of the related dopamine transporter (DAT). Here we show that MKP3 decreases PKC-mediated down regulation of NET expressed in PC12 cells. We demonstrate that this process involves a PKC-stimulated decrease of NET surface expression that is dependent on dynamin. Surprisingly, MAP kinase inhibitors have no effect on the PKC-mediated regulation of NET activity, suggesting that, like PKC-mediated regulation of the DAT, the acute activation of MAP kinases is not likely to be involved. To elucidate potential mechanisms we used a substrate trap-based assay to identify extracellular-signal-regulated kinase (ERK)1/2 as the predominant substrate of MKP3. Furthermore we also established that brief chemical stabilization of a modified destabilized MKP3 does not alter PKC-mediated down regulation of NET. Finally, the expression of a dominant negative version of H-Ras, an upstream activator of ERK1/2, abolishes phorbol 12-myristate 13-acetate (PMA)-mediated down regulation of NET in a manner similar to MKP3. Taken together we propose that chronic MKP3 expression regulates surface NET through the sustained inhibition of ERK1/2 MAP kinase signaling that alters gene expression in PC12 cells. This is supported by gene expression data from naïve and MKP3-expressing PC12 cells that reveal robust decreases in gene expression of several genes in the MKP3-tranfected cells. Interestingly, caveolin-1, a protein with a critical role in membrane protein trafficking is down regulated by MKP3 expression. We further show that selective silencing of the caveolin-1 gene in naïve PC12 cells attenuates PKC-mediated downregulation of NET activity, consistent with a potential role for caveolins in regulating NET surface expression. In summary, these results suggest that chronic MKP3 expression alters the expression of genes in PC12 cells that are involved in the regulation of NET surface expression.

## Introduction

The norepinephrine transporter (NET) belongs to the SLC6A family of Na/Cl-dependent neurotransmitter transporters (Kristensen et al., 2011). NET is responsible for the clearance of the neurotransmitters dopamine (DA) and norepinephrine (NE) following synaptic release from both central and peripheral terminals. It is a crucial determinant for the termination and regulation of the transmitter signal, and is also responsible for the recycling of the neurotransmitter back into the presynaptic neuron. Altered NET activity has been associated with mood disorders and cardiovascular diseases and a functional polymorphism causing the loss of NET function has been found in patients with orthostatic intolerance (Shannon et al., 2000). NET knockout mice display excessive tachycardia and are hypersensitive to psycho stimulants like cocaine (Xu et al., 2000; Keller et al., 2004). The fact that psychostimulants, classic tricyclic antidepressants (TCAs), and newer more selective antidepressants target and inhibit NET provides a compelling rationale for understanding the function and regulation of the NET (Kristensen et al., 2011). Additionally duloxetine a SERT/NET inhibitor is now used in the treatment of neuropathic pain.

Like most members of the Na/Cl-dependent carrier family, NET is expressed at the plasma membrane, where it catalyzes the accumulation of NE from the extracellular space (Torres et al., 2003; Kristensen et al., 2011). To translocate NE from the extracellular space NET must be targeted to the cell surface, but studies have found the expression of NET in some areas of the prefrontal cortex is predominantly intracellular (Miner et al., 2003). In further experiments it was established that intracellular NET can be recruited to the cell surface in states of high neuronal activity (Miner et al., 2006) and depolarization results in increased NET activity (Mandela and Ordway, 2006). This result implies that the intracellular pools of NET act as a reserve pool and surface expression of NET is regulated. Understanding the cell biology that is involved in this regulation of NET function and surface expression could provide important insights into our understanding of catecholamine physiology.

Previous studies of NET regulation in PC12 cells have demonstrated that protein kinase C (PKC) activation decreased NET activity (Uchida et al., 1998; Mandela and Ordway, 2006). The mechanism of this regulation was not established but the results of one study implied that changes in surface expression might be involved (Mandela and Ordway, 2006). In the present study we have investigated the mechanism of PKC-mediated regulation of NET further and have tested the involvement of MAP kinase phosphatase 3 (MKP3 or DUSP6) in the modulation of endogenously expressed NET in PC12 cells. This was triggered by our previous study on the closely related dopamine transporter (DAT) in which we found that PKC-mediated and dynamin-dependent internalization of the DAT was attenuated by MKP3 (Mortensen et al., 2008). MKPs are dual specificity phosphatases and they inactivate MAP kinases by dephosphorylating both threonine and tyrosine residues (Camps et al., 2000; Farooq and Zhou, 2004; Dickinson and Keyse, 2006; Kondoh and Nishida, 2007; Caunt and Keyse, 2013). PC12 cells are derived from a rat pheochromocytoma and display characteristics of chromaffin and sympathetic neuron cell precursors. Furthermore PC12 cells adopt a neuronal-like morphology when treated with nerve growth factor (NGF) and are therefore useful for studying relevant molecular noradrenergic physiology. PC12 cells have also been used as a model system to study mechanistic aspects of DAT regulation (Melikian and Buckley, 1999; Loder and Melikian, 2003; Holton et al., 2005; Boudanova et al., 2008; Navaroli and Melikian, 2010; Navaroli et al., 2011; Gabriel et al., 2013). Here we show PKC-mediated regulation of NET activity in PC12 cells can be modified by over-expressing MKP3. We also provide evidence that the PKC-induced down regulation is not caused by an immediate short-lived downstream activation of MAP kinase pathways, and we provide evidence that suggest a process involving sustained inhibition of MAP kinases including extracellular-signal-regulated kinase (ERK)1/2 resulting in changes in gene expression that mediate the observed regulation of NET.

## Materials and Methods

### Materials

[^3^H]-DA was obtained from NEN (Boston, MA, USA). Reagents for uptake and binding buffers, uptake inhibitors and substrates were from Sigma-RBI (St. Louis, MO, USA). Cell culture media, MEM, FCS, horse serum, penicillin/streptomycin, glutamine and D-PBS were from Invitrogen Life Technologies (Carlsbad, CA, USA). All other reagents were of analytical grade or better.

### Molecular Biology

For expression and production of stable MKP-expressing PC12 cell lines MKP3 was subcloned into pIRES2 to enable color selection and stably expressing colonies were isolated in the presence of G418 (1 mg/ml). Stable expression of HA-tagged NET was achieved by blasticidin (5 μg/ml) selection of colonies stably expressing an IRES construct co-expressing HA-tagged NET and blasticidin-S deaminase. Point mutations were generated using the QuikChange Site-Directed Mutagenesis Kit according to the manufacturer’s protocol (Stratagene Cloning Systems, La Jolla, CA, USA). The expression construct for expressing the dominant negative RasN17 mutant was a kind gift from Dr. Philip Stork, Oregon Health and Science University. The ProteoTuner system including vectors and the Shield1 compound for destabilizing MKP3 expression was obtained from Clontech (Mountain View, CA, USA). The destabilizing domain was attached to the C-terminus of MKP3.

### Uptake Assays

Naïve and stable PC12 cells described above were maintained in Dulbecco’s modified Eagle’s medium supplemented with 10% fetal bovine serum supplemented with penicillin and streptomycin and for stable cell lines G418 (1 mg/ml) and/or blasticidin (5 μg/ml) in a humidified atmosphere with 5% CO_2_ at 37°C. The cells were preincubated with or without phorbol 12-myristate 13-acetate (PMA) for 30 min. Uptake assays of PC12 cells were carried out in PBS containing 1 mM MgCl_2_, 0.1 mM CaCl_2_ and 10 μM RO41-0960. RO41-0960 is a catechol-O-methyl transferase inhibitor that prevents the degradation of catecholoamines. Uptake assays were performed at room temperature for 10 min with substrate concentrations between 50 nM and 100 nM. Uptake was terminated by two washes and cells were lysed and counted on a liquid scintillation counter. NET-specific uptake was determined by subtracting mock-transfected cells or subtracting background uptake obtained in the presence of NET inhibitor desipramine (100 μM).

### Cell Surface Biotinylation

After drug treatment cells were washed and incubated with 2 mg/ml sulfo-NHS-SS-biotin (Pierce, Rockford, IL, USA). The cells were quenched with 100 mM glycine buffer, washed with PBS, and lysed in lysis buffer (50 mM Tris, pH 7.5, 150 mM NaCl, 5 mM EDTA and 1% Triton X-100 and protease inhibitor cocktail; Roche Applied Science). The cell lysate was incubated on ice and centrifuged at 14,000× *g* before incubation with NetrAvidin Resin (Pierce) overnight at 4°C. The beads were separated from the supernatant by centrifugation at 5000× *g* for 15 min, washed three times with lysis buffer, twice with a high-salt wash buffer, and once with a no-salt wash buffer. Proteins were separated on SDS-PAGE gels and immunoblotted. Expression of HA-tagged NET was probed with a HA antibody from Covance (Princeton, NJ, USA). Imaging and band intensity analysis was performed on a LI-COR Odyssey imaging system.

### Biochemistry

To examine the ubiquitylation of HA tagged NET, cells were lysed following drug treatments in ice cold lysis buffer (50 mM Tris pH 7.5, 150 mM NaCl, 10% glycerol, 1% Triton X-100, 10 mM N-ethyl-maleimide and protease inhibitor cocktail). Cell lysates were immunoprecipitated with a HA specific antibody Roche Applied Science (Indianapolis, IN, USA) and samples were separated on SDS-PAGE gels and immunoblotted. Ubiquitylated NET was detected using a monoclonal antibody to ubiquitin (P4D1) from Santa Cruz Biotechnology (Santa Cruz, CA, USA). NET was detected with a HA antibody from Covance (Princeton, NJ, USA). For other types of immuno-blotting the protein samples were separated on SDS-PAGE gels and immunoblotted. Imaging and band intensity analysis was performed on a LI-COR Odyssey imaging system. Antibodies from Cell Signaling Technology were used to detect the level of and the activation state of pERK1/2 (9101 and 9102). To detect levels of caveolin-1 expression antibodies from Santa Cruz Biotechnology was used (N-20, sc-894). To detect levels of caveolin-2 expression antibodies from Abcam (Cambridge, MA, USA) was used (ab2911).

### Substrate Trapping Experiments

*E. coli* Bl21(DE3) transfected with vector containing MKP3 with a carboxy terminal 12xHis tag were allowed to grow overnight, diluted 20 fold the next morning and allowed to reach OD600 of 0.6–0.8. 0.2 mM IPTG was added to induce MKP3 expression and the culture incubated at room temperature with shaking for 4 h. The culture was harvested by centrifugation and resuspended in TBS (50 mM Tris pH 8, 150 mM NaCl, 10 mM imidazole) containing protease inhibitors and 1 mg/mL lysozyme. Incubtion was continued for 10 min on ice followed by sonication and addition of 1% Triton X-100. After 10 min incubation on ice, debris was pelleted by spinning at 16,000 *g* for 20 min at 4°C. The lysate was incubated with washed Ni-NTA agarose beads (Qiagen, Valencia, CA, USA) and incubated for 1 h at 4°C, washed twice in TBS containing 20 mM imidazole and 1% Triton X-100 and twice in TBS with 20 mM imidazole. MKP3 was eluted from the Ni-NTA agarose beads in TBS with 250 mM imidazole and 1 mM tris(2-carboxyethyl)phosphine.

PC12 cells were treated for 30 min at 37°C with 1 mM Na_3_VO_4_, 1 mM H_2_O_2_ and 1 μM PMA. Cells were lysed in lysis buffer (20 mM Tris pH 8, 100 mM NaCl, 0.1 mM EDTA, 1% Triton X-100, 10 mM imidazole, 1 mM iodoacetic acid and protease inhibitors) for 30 min at 4°C. 10 mM 2-ME and 0.2 mM DTT were added and incubation continued for 15 min at 4°C to quench unreacted iodoacetic acid. The resulting lysate was centrifuged at 16,000 *g* for 20 min, 4°C. The lysate was precleared by incubating for 1 h at 4°C with washed Ni-NTA beads. The precleared lysate was incubated overnight with Ni-NTA agarose beads pre-conjugated to the *E. coli* expressed MKP3. The next day the beads were washed repeatedly in lysis buffer followed by incubation with 8M urea to release MKP3 associated proteins but allow MKP3 to remain bound to the Ni-NTA agarose beads. The eluate was precipitated with acetone, resuspended in SDS-PAGE loading buffer and prepared for gel electrophoresis. The samples were run on 4%–12% NuPAGE Bis-Tris gels (Invitrogen), stained with Coomassie stain or subjected to western blotting with antibodies directed against phospho-ERK1/2 (Santa Cruz Biotechnology) or phospho-Tyrosine (Santa Cruz Biotechnology) using standard laboratory techniques.

### DNA Microarray

Total RNA from PC12 cells was isolated and purified using RNeasy columns (Qiagen). Probes for hybridization to the DNA microarrays were prepared from 5 μg of total RNA according to Affymetrix protocol. The probes were hybridized to the Affymetrix Rat Genome 230 2.0 arrays and washed and stained with streptavidin-phycoerythrin according to Affymetrix protocol on an Affymetrix fluidics station. The arrays were finally scanned on an Affymetrix GeneChip scanner. Average intensities were scaled to a target intensity of 200 to compensate for differences in fluorescence intensities and to enable comparisons between arrays. Affymetrix’s microarray analysis suite (MAS 5.0 statistical algorithm) was used to analyze the data.

### shRNA

To produce Caveolin-1 knock down cell lines PC12 cells were transfected with the MISSION^TM^ shRNA plasmid TRCN0000112660 (Cav-1-60) or TRCN0000112661 (Cav-1-61) (Sigma Aldrich, St. Louis, MO, USA) which produces an shRNA that targets the coding region of Caveolin-1. Stably expressing cells were selected using 5 μg/ml puromycin.

## Results

### PKC-Regulated NET Surface Expression Is Dynamin Dependent

**Figure 1:**
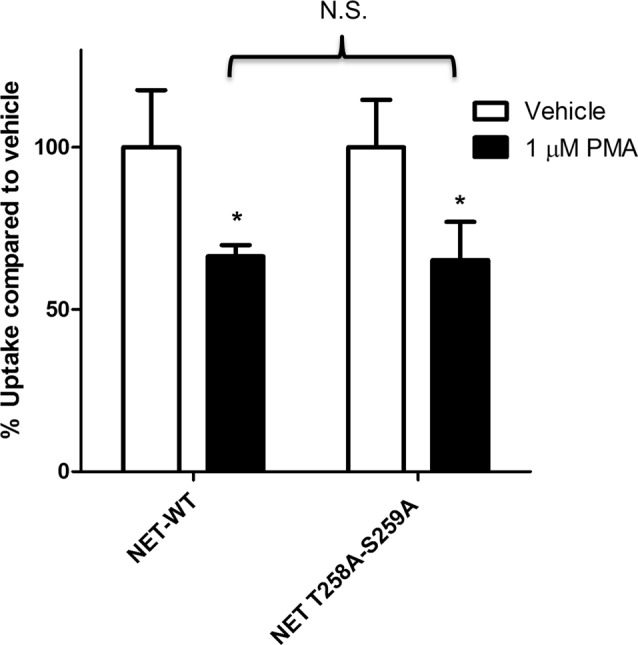
Protein kinase C (PKC)-mediated down regulation of norepinephrine transporter (NET) activity in PC12 cells is not dependent on phosphorylation of residues 258 and 259. Naïve PC12 cells were transiently transfected for 48 h with cDNAs for WT-NET or T258A/S259A-NET. Radiolabeled uptake was measured following 30 min incubation with PKC activator phorbol 12-myristate 13-acetate (PMA; 1 μM) subtracting mock-transfected cells. All experiments were performed in triplicate in three independent experiments. Both cDNAs were significantly affected by PKC activation (*p < 0.05) but no significant difference (N.S.) in the magnitude of the PKC-mediated downregulation of uptake was observed between the two cDNAs (two-way ANOVA with Bonferroni *post hoc* analysis).

Earlier studies have shown that PKC activation results in decreased NET activity in PC12 cells (Uchida et al., 1998; Mandela and Ordway, 2006). Figure 1 shows that application of 1 μM PMA to PC12 cells transiently expressing the NET results in a 30%–40% decrease in NET activity. Because a previous study showed that direct phosphorylation of NET could affect NET activity in human placental trophoblast cells (Jayanthi et al., 2006) we next examined the effect of PKC on a double mutant of NET in which PKC-mediated NET phosphorylation sites were eliminated (hNET-T258A/S259A). In PC12 cells we found that the activity of the hNET-T258A/S259A phosphorylation site mutant was down regulated to a similar degree as the wild type transporter following PKC activation (Figure 1) thus indicating that direct phosphorylation of T258 and S259 in NET is not required for the reduction in uptake observed after PMA treatment.

Because phosphorylation of T258 and S259 does not appear to be involved in the down regulation of NET activity we speculated that other post-translational modifications such as ubiquitinylation could be responsible for the effect on transport activity. It has previously been observed that DAT is ubiquitylated following activation of PKC and that ubiquitylation is necessary for the internalization of DAT (Miranda et al., 2005, 2007; Mortensen et al., 2008). To test whether NET is ubiquitylated following PKC activation we transiently transfected PC12 cells with a HA-tagged version of NET and immunoprecipitated the transporter following drug treatment. We used an antibody against ubiquitin to determine if NET is ubiquitylated in vehicle and phorbol ester-treated conditions. In contrast to what was found with DAT we observed no basal or activated ubiquitylation of NET (Data not shown). In summary these results suggest that phosphorylation and ubiquitylation is not involved in the PKC-mediated regulation of NET in PC12 cells.

**Figure 2:**
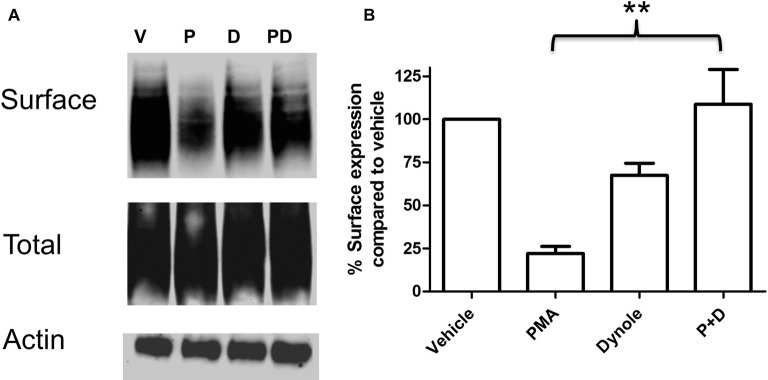
PKC activation cause a dynamin dependent reduction in surface expression of NET. PC12 cells stably transfected with an HA-tagged NET were treated with PKC activator PMA and dynamin inhibitor Dynole 34-2. Cell surface biotinylation was performed and surface expressed NET was visualized through immunoblotting **(A)**. Band densities were quantified from three independent experiments **(B)**. Statistical significance was determined employing one-way ANOVA with Bonferroni *post hoc* analysis (**P < 0.01). P:PMA (1 μM), D:Dynole 34-2 (20 μM).

Multiple studies support the idea that the effects of PKC activation on monoamine transporter activity are mediated through trafficking events involving endocytosis of carrier proteins from the cell surface (Mortensen and Amara, 2003). Analyses of the saturation kinetic of NE uptake into PC12 cells demonstrated that PKC activation does not alter apparent *K*_t_, but displays a decrease in *V*_max_ (Table 1) a result consistent with a reduction in the number of surface transporters. To further investigate the mechanism of this regulation we produced a stable cell line with NET tagged N-terminally with an HA-tag. This was required as a result of the low levels of endogenously expressed NET in PC12 cells that makes endogenous NET difficult to detect in biochemical assays. In agreement with the uptake assays, cell surface biotinylation assays showed decreased surface expression of HA-NET following PKC activation (Figure 2 and Supplementary data, Figure S1). To further explore the mechanism we took advantage of a newly developed pharmacologic reagent, Dynole 34-2, that inhibit dynamin (Hill et al., 2009). Dynamin is intrinsically involved in several processes of membrane internalization and has been suggested to be involved in monoamine transporter trafficking (Conner and Schmid, 2003; Kristensen et al., 2011). To investigate if dynamin is involved in the PKC-mediated regulation of NET activity and cell surface expression, we performed cell surface biotinylation assays in the presence or absence of 20 μM Dynole 34-2. In support of a role for dynamin-dependent events in regulating NET surface expression, the dynamin inhibitor attenuates the decrease in NET surface expression following PKC activation (Figure 2 and Supplementary data, Figure S1).

**Table 1:** Norepinephrine transporter (NET)-specific uptake kinetics in naïve and mitogen-activated protein kinase 3 (MKP3)-expressing PC12 cells following protein kinase C (PKC) activation.

	PC12 Veh	PC12 1 μM PMA	PC12-MKP3 veh	PC12-MKP3 1 μM PMA
*V*_max_/%	100	53 ± 4	100	77 ± 10*
K_t_/nM	180 ± 3	160 ± 3	410 ± 7^##^	420 ± 13^##^

Radiolabeled uptake was measured in naive and MKP3-expressing cells following 30 min incubation with PKC activator PMA (1 μM) or vehicle subtracting mock-transfected cells. Statistical significance was determined employing two-way ANOVA with Bonferroni post hoc analysis. All experiments were performed in triplicate in three independent experiments (*p < 0.05 comparing V_max_ following PMA treatment, ^##^*p* < 0.01 comparing effect of MKP3 expression on K_t_).

### MKP3 Modulates PMA Induced Down Regulation of NET in PC12 Cells

**Figure 3:**
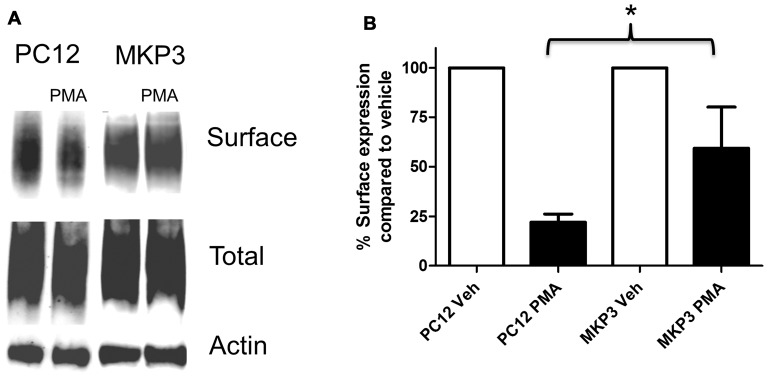
PKC-mediated decrease in NET surface expression is attenuated by MAP kinase phosphatase 3 (MKP3). Cell surface biotinylation was performed on naïve (PC12) and MKP3-expressing (MKP3) PC12 cells following PKC activation by PMA (1 μM) for 30 min. NET surface expression was visualized by immunoblotting **(A)**. Band densities were quantified from three independent experiments **(B)**. Statistical significance was determined employing two-way ANOVA with Bonferroni *post hoc* analysis (*p < 0.05 comparing surface expression following PMA treatment).

Previously, we described the expression cloning of the MKP3 as a protein that regulates the function of the DAT (Mortensen et al., 2008). To establish whether this effect was specific to the DAT or also extended to other related neurotransmitter transporter we examined PKC-mediated regulation of the NET in PC12 cells stably expressing MKP3 (Figure 3). We found that, as we had observed with DAT, MKP3 attenuates the PMA-induced down regulation of endogenous NET activity (Table 1). Importantly, the PKC-mediated decrease in *V*_max_ was reduced when MKP3 was expressed in the PC12 cells. Interestingly, we also observed a significant increase of the *K*_t_ in the presence of MKP3. Although the amount of endogenous surface transporter could not be assessed using cell-surface applied biotin-reagents and antibody detection, we were able to use cell surface biotinylation assays on PC12 cells expressing exogenous NET constructs. In these experiments, we consistently observed an attenuation in the decrease in NET surface expression following PKC activation as a result of MKP3 expression (Figure 3) demonstrating that MKP3 regulates the dynamin-dependent trafficking of NET.

### MAP Kinase Activation Does Not Correlate with Internalization

**Figure 4:**
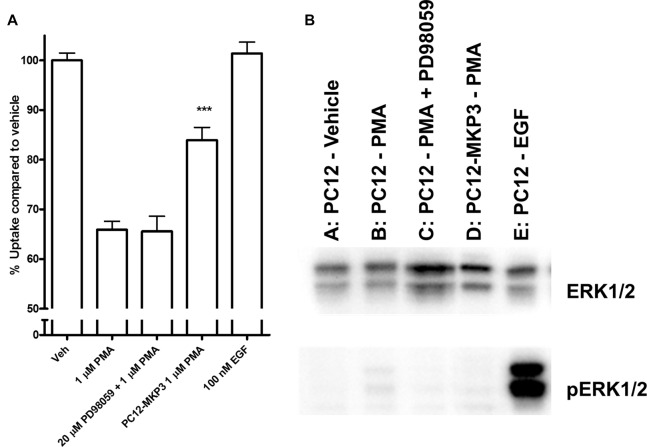
Downstream activation of extracellular-signal-regulated kinase (ERK)1/2 does not mediate the effect of PKC activation on NET activity. Naïve or MKP3-expressing (PC12-MKP3) PC12 cells were pretreated for 30 min with PKC activator PMA (1 μM), MEK1/2 inhibitor PD98056 (20 μM), or EGF (100 nM) and NET-specific radiolabeled uptake was performed subtracting desipramine insensitive background uptake **(A)**. All experiments were performed in triplicate in three independent experiments. Statistical significance was determined employing one-way ANOVA with Bonferroni *post hoc* analysis (***p < 0.001 comparing effects of PMA treatment. There was no significant effect of PD98056 on the effect of PMA treatment and no significant effect of EGF treatment compared to vehicle). Identical pharmacologic treatments of the same cell types were performed and ERK1/2 map kinase activation was assayed by immunoblotting **(B)**. PMA showed small activation of ERK1/2 and this was inhibited by PD98056. EGF showed strong activation of ERK1/2.

Because the MAP kinases are the primary target of MKPs, we investigated whether MAP kinases are a downstream target of the signaling cascade activated by PMA and whether their activation is directly linked to inhibition of NET activity. We investigated the activation state of various MAP kinases following PMA treatment in either absence or presence of MKP3 in PC12 cells endogenously expressing NET (Figure 4). As we found for DAT regulation (Mortensen et al., 2008) MAP kinase inhibitors did not block the downregulation of surface NET following PKC activation. PMA treatment of PC12 cells did induce a weak phosphorylation and activation of ERK1/2 (Figure 4), but did not activate the stress-induced JNKs or p38 mitogen-activated protein kinase (MAPKs; Data not shown). The phosphorylation and activation of ERK1/2 was abolished when MKP3 was stably expressed in PMA treated PC12 cells. To test whether the transient activation of ERK1/2 was sufficient to produce the down regulation of NET by PKC, we examined the effects of PD98059, an inhibitor of the upstream MAP kinase kinases MEK1/2. As expected, pretreatment of cells with PD98059 decreased PMA-induced activation of ERK. However, PD98059 did not prevent PMA-induced down regulation of NET activity (Figure 4) indicating that transient activation of the ERK1/2 pathway is not likely to be involved in the decrease in NET surface expression. Consistent with this lack of an effect of the ERK1/2 pathway, acute application of EGF, a potent activator of ERK1/2, had no effect on transport activity. Similarly, inhibition of JNKs with SP600125 (50 μM) or p38 MAPKs with SB203580 (10 μM) or MEK1/2 with U0126 (20 μM) could not prevent the PMA induced down regulation of NET activity (data not shown).

### Identification of ERK1 and ERK2 as the Major Substrates for MKP3

**Figure 5:**
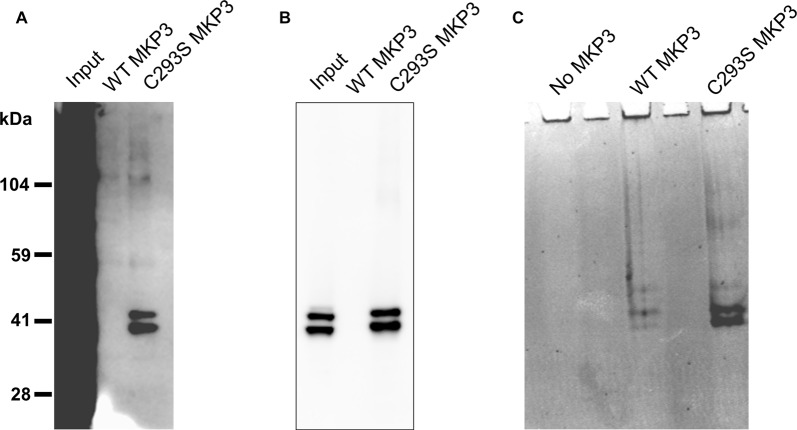
Substrate trapping reveals ERK1/2 as the major substrates for MKP3. PC12 lysate was incubated with WT MKP3 or substrate trapping mutant C293S MKP3, followed by isolation of MKP3 and elution of co-precipitated proteins. Eluted proteins were subjected to western blotting with **(A)** phospho-tyrosine antibody, **(B)** phospho-ERK1/2 antibody or the gel was stained with Coomassie stain **(C)**. The blots show that MKP3 efficiently captures ERK1/2 but does not reveal other kinases or proteins to be substrates for MKP3.

Because we were not able to directly implicate classical MAP kinases in the observed regulation of NET activity, we speculated that MKP3 was dephosphorylating a novel substrate distinct from ERK1/2 that could mediate the effects of PKC activation. To identify substrates of MKP3 in PC12 cells, we used a pulldown assay in which His-tagged WT MKP3 or mutant C293S MKP3 were incubated with PMA-activated PC12 cell lysate and isolated with Ni-NTA agarose beads (Flint et al., 1997; Blanchetot et al., 2005). Normally, the interaction between MKP3 and substrate is of a transient nature. However, when the MKP3 active site residue C293 is mutated to serine (C293S) the interaction between MKP3 and phosphorylated substrate is stabilized creating a “substrate trap” that enables the isolation of MKP3 substrates by affinity purification. As shown in Figure 5A, PC12 lysates contain numerous tyrosine-phosphorylated proteins when assessed with an anti-phosphotyrosine antibody. Affinity purification of proteins using WT MKP3 identifies modest background levels of tyrosine-phosphorylated proteins. On the other hand, when employing the same affinity purification assay with the C293S MKP3 variant two tyrosine phosphorylated proteins with molecular weights close to 41 kDa preferentially associates with the substrate-trapping mutant (Figure 5A, lane 3). Because MKP3 is an ERK1/2 phosphatase, we hypothesized that ERK1 and ERK2 represent the two proteins associated with C293S MKP3. Immuno-blotting with a phospho-ERK1/2 antibody confirmed the identity of the two bands to be ERK1 and ERK2 (Figure 5B). The samples were also stained with Coomassie protein stain in order to identify interacting proteins that might not be recognized by the phosphotyrosine antibody (Figure 5C). Coomassie staining likewise identified two prominent interacting proteins with ~41 kDa molecular weight for the C293S MKP3 substrate trapping mutant, as well as much weaker bands of the same size proteins interacting with WT MKP3. Mass spectrometry analysis of these two proteins corroborated the identity of the two interacting proteins to be ERK1 and ERK2, respectively (data not shown). No other protein species were identified consistently in affinity purifications using the substrate trap. In conclusion, ERK1 and ERK2 are the substrates for MKP3 and there do not appear to be other major substrates in PC12 cells.

### Long Term Inactivation of ERK1/2-Mediated Signaling Is Necessary for Regulating NET Activity

**Figure 6:**
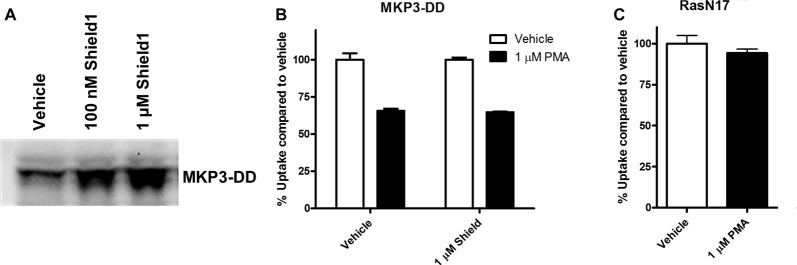
Modulation of PKC-mediated regulation of NET activity is dependent on long term processes. A destabilized version of MKP3 (MKP3-DD) was stably expressed in PC12 cells. Short term stabilization of this destabilized MKP3 was achieved pharmacologically by a 4-h incubation with the Shield1 compound **(A)**. Following restoration of the MKP3 protein by Shield1 (1 μM for 4 h) PKC was activated by PMA for 30 min and NET-specific radiolabeled uptake was performed as above comparing with vehicle treated control **(B)**. All experiments were performed in triplicate in three independent experiments. No significant attenuation of PKC-mediated down regulation of NET was observed following MKP3 protein restoration (two-way ANOVA with Bonferroni *post hoc* analysis). A dominant negative mutant of the ras protein (RasN17) was stably expressed in PC12 cells and PKC was activated by PMA for 30 min and NET-specific radiolabeled uptake was performed as above comparing with vehicle treated control **(C)**. All experiments were performed in triplicate in three independent experiments. PKC mediated down regulation was completely inhibited as there was no significant difference between vehicle and PMA treated cells (Student’s *t*-test).

The experiments employing map kinase inhibitors argue against an acute involvement of ERK1/2 in the regulation of NET activity and our evidence that ERK1/2 are the major cellular substrates of MKP3 suggest that the actions of MKP3 on NET activity could be a result of more downstream effects of the ERK1/2 pathway linked to gene expression. To test whether long term changes in gene expression are responsible for the effects of MKP3 we designed two different experiments (Figure 6). In one experiment we took advantage of a method that allows MKP3 activity to be regulated by attaching a destabilizing domain that normally targets the protein for degradation (DD; Banaszynski et al., 2006; Egeler et al., 2011). Incubation of the cells with a stabilizing compound called Shield1 prevents degradation and maintains the activity of the protein (MKP3), We found a 4-h incubation with 1 μM Shield1 dramatically increased the levels of detectable MKP3 expression in PC12 cells expressing the destabilized version of MKP3 (MKP3-DD; Figure 6A). Importantly, we observed PKC-mediated down regulation of NET activity when MKP3-DD expressing cells were incubated either in the absence or the presence of the stabilizing drug for 4 h (Figure 6B). This observation suggests that the stabilization of MKP3 activity for less than 4 h is not sufficient to alter PKC-activated internalization of the NET. In another experiment we used another approach to target the ERK1/2 kinase signaling pathway genetically by expressing RasN17 in PC12 cells. RasN17 is a dominant negative version of H-Ras that is a major upstream activator of ERK1/2 kinase signaling. This mutant has been extensively studied and used as a tool to inactivate the ERK1/2 MAP kinase signaling pathway (Chen et al., 2001). In the RasN17 expressing cells, we observed no PKC-mediated down regulation of NET activity in these cells, an effect that appears even stronger than the reduction in NET down regulation seen in MKP3 expressing cells (Figure 6C).

### MKP3 Expression Produces Dramatic Changes in Gene Expression

These results indicate that the regulation of NET surface expression depends upon a more chronic action of MKP3 activity, and thus, we used DNA microarrays to examine how the expression of various genes might be altered by MKP3 expression. RNA was isolated from naïve and MKP3-expressing cells and analyzed with Affymetrix GeneChip Rat Genome 230 2.0 Arrays. RNA was isolated from naïve and MKP3 expressing PC12 cells at several passages and gene expression patterns were compared. We observed variations of up to several fold in gene expression within the same cell type. This observation made it necessary to use cells at comparable growth state and passage and to employ stringent selection criteria to examine genes that displayed very robust changes in gene expression between naïve and stable MKP3-expressing cells. Using selection criteria requiring changes higher than 10-fold we identified several 100 genes that were differentially expressed when MKP3 was expressed in PC12 cells (Supplementary data, Data Sheet 1). Not unexpectedly, we found most of the genes were down regulated in the cells where ERK1/2 signaling is reduced by MKP3 expression. Several of these genes have already been shown to be regulated by ERK1/2 dependent signaling (Chen et al., 2001). We have included a list of the top 20 genes that all show more than 50-fold decreases in expression levels in the cells expressing MKP3 (Table 2).

**Table 2:** Global gene expression is significantly affected by MKP3 expression.

Probeset ID	Genetitle	Expression level (naïve PC12) Arbitrary unit (Average is 200)	Log ratio	Fold change
1387138_at	tachykinin 2	3005	5.7	52.0
1393783_at	similar to contactin associated protein-like 2a	229	5.7	52.0
1371527_at	epithelial membrane protein 1	1803	5.8	53.8
1393281_at	caveolin 1	1003	5.8	53.8
1370562_at	calcitonin-related polypeptide, beta	1455	5.9	59.7
1372190_at	aquaporin 4	225	6.1	66.3
1374235_at	regulator of calcineurin 2	180	6.1	66.3
1384262_at	protein phosphatase 1, regulatory subunit 3B	920	6.3	76.1
1370907_at	beta-galactosamide alpha-2,6-sialyltranferase 1	790	6.3	78.8
1377642_at	caveolin 2	490	6.4	81.6
1398398_at	Homeo box A10	1232	6.4	84.4
1387316_at	chemokine (C-X-C motif) ligand 1	1457	6.6	93.7
1370650_s_at	bradykinin receptor B2	329	6.6	93.7
1386890_at	S100 calcium binding protein A10	5145	6.8	111.4
1370247_a_at	peripheral myelin protein 22	7057	6.9	119.4
1370334_at	pleckstrin homology domain containing (evectins)	4960	7.1	132.5
1375170_at	S100 calcium binding protein A11 (calizzarin)	3233	7.2	142.0
1367614_at	annexin A1	7899	7.2	147.0
1367949_at	proenkephalin	2983	7.5	181.0
1369814_at	chemokine (C-C motif) ligand 20	5677	7.7	207.9

*RNA was isolated from naïve and MKP3 expressing cells and analyzed with Affymetrix GeneChip Rat Genome 230 2.0 Arrays. Affymetrix’s microarray analysis suite (MAS 5.0 statistical algorithm) was used to analyze the data. Expression level in arbitrary units with 200 being the expression level of an average gene and the log ratio difference in expression between the two cell lines reported by the software is displayed. The log ratio was converted to fold change by the formula: 2^(log ratio)^*.

### Caveolin-1 Silencing Regulates NET Activity

**Figure 7:**
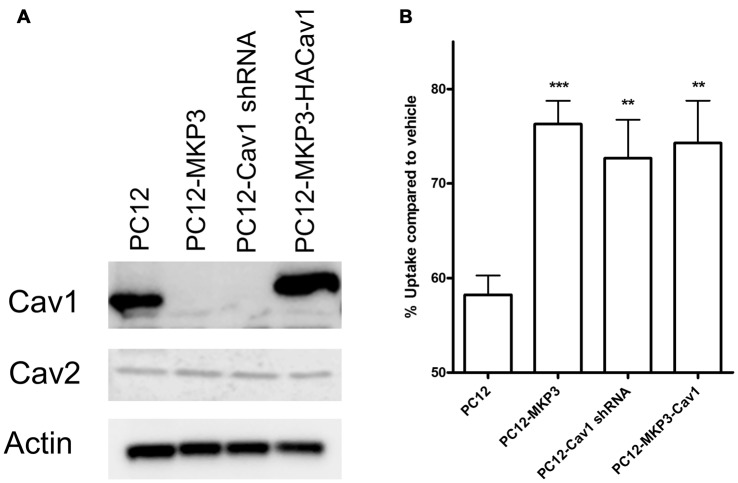
Knockdown of caveolin-1 attenuates PKC-mediated down regulation of NET function. Immunoblotting demonstrate silencing of caveolin-1 expression by stable expression of Cav1 shRNA construct (PC12-Cav1 shRNA) or MKP3 (PC12-MKP3) and successful re-introduction of caveolin-1 in MKP3 expressing cells (PC12-MKP3-HACav1) **(A)**. Following Cav1 silencing or re-introduction PKC was activated by PMA for 30 min and NET-specific radiolabeled uptake was performed as above **(B)**. Results are displayed as percentage uptake following PMA treatment compared to vehicle treated control of respective cell type. Expression of MKP3 or silencing of Caveolin1 by the shRNA construct attenuates NET downregulation following PKC activation to a similar degree **(B)**. Re-introduction of caveolin1 into MKP3 expressing PC12 cells does not recover the PKC-mediated down regulation **(B)**. All experiments were performed in triplicate in three independent experiments. Results from three independent experiments were analyzed by two-way ANOVA with Bonferroni *post hoc* analysis (**p < 0.01, ***p < 0.001 comparing effects of PMA treatment. There was no significant difference in effect of PMA between MKP3 and MKP3-Cav1 cells).

Several of the genes identified in DNA microarrays had already been implicated in neurotransmitter transporter regulation and one of these, caveolin-1, was robustly silenced by MKP3 expression (Table 2). Caveolins are the principal components of caveolae that were previously pharmacologically implicated in the regulation of NET activity (Jayanthi et al., 2004). To investigate if MKP3-mediated down regulation of caveolin-1 expression was involved in the regulation of NET activity we specifically silenced the expression of caveolin-1 in parent PC12 cells. Silencing of caveolin-1 expression was achieved by stably expressing different shRNA constructs. Stable cell lines from two different shRNA (cav-1-60 and cav-1-61) constructs were evaluated and both showed comparable inhibition of caveolin-1 protein content. The expression of these constructs achieved a reduction of caveolin-1 expression similar to what was observed in the MKP3-expressing PC12 cells (Figure 7A). Importantly the sensitivity of NET activity to PKC-mediated regulation was altered to a similar degree in the two knock-down cell lines and the MKP3-expressing cells suggesting the down regulation of the caveolin-1 protein by MKP3 could contribute to the observed alterations in trafficking and decrease in NET activity (Figure 7B). However, we could not rescue the effect of MKP3 expression by re-introducing caveolin-1 expression in the MKP3 expressing PC12 cells as the PKC-mediated down regulation was still attenuated (Figures 7A,B, the higher molecular weight of the reintroduced caveolin-1 is due to an added tag). This result suggests that down regulation of caveolin-1 expression is not the sole mediator of MKP3’s effects and implies that rescue of the PKC-mediated effect may require the introduction of other MKP3 down regulated genes in addition to caveolin-1. Another potential gene candidate, caveolin-2, was also examined, However, additional studies showed that the protein expression data for caveolin-2 did not correlate with the data from the microarray experiment as we found that caveolin-2 expression was unaltered by MKP-3 expression or caveolin-1 shRNA expression (Figure 7B). We conclude that MKP3-mediated down regulation of only caveolin-1 and not caveolin-2 is required for regulating NET surface activity.

## Discussion

Using a genetic complementation strategy, we previously identified the dual-specificity MKP3, as a protein responsible for cell-type specific differences in the PKC-mediated down regulation of the human DAT (Mortensen et al., 2008). In Xenopus oocytes expressing the DAT, activation of PKC using PMA produces a dramatic internalization of DAT from the cell surface (Zhu et al., 1997). However, in other heterologous systems, such as the SK-N-SH cell line, we observed little or no PKC-dependent down regulation of the DAT. Co-expression of the DAT with a cRNA library from the SK-N-SH cell line blocked PKC-mediated DAT internalization and using a sequential pooling and division strategy of the cRNA library we isolated the MKP3 (DUSP6 or PYST1) as a protein that could attenuate the PKC-activated down regulation of DAT function. In the present study, we now show that MKP3 also can attenuate PMA induced regulation of the related NET in neuroendocrine PC12 cells endogenously expressing NET. We furthermore explore potential mechanisms for how MKP3 produces its effects on PKC-mediated NET trafficking.

MKPs are dual specificity phosphatases that inactivate MAP kinases by dephosphorylating a threonine and a tyrosine residue (Kondoh and Nishida, 2007; Caunt and Keyse, 2013). MAP kinases have previously been shown to regulate the activity of neurotransmitter transporters. However, our studies are the first to illuminate the role of MKP3 in these regulatory pathways. The p38 MAP kinase has been proposed to mediate the insulin-dependent up regulation of intrinsic NET activity in SK-N-SH cells (Apparsundaram et al., 2001) and has been show to increase the delivery of serotonin transporters to the cell membrane (Samuvel et al., 2005). Inhibition of ERK1/2 MAP kinases leads to a small reduction in DAT activity believed to be a result of internalization (Morón et al., 2003) and DA receptor-mediated regulation of the DAT has also been linked to the MEK/ERK pathway (Bolan et al., 2007). All these studies examine the acute effects of inhibitors of MAP kinase signaling that show no effects in our studies, and thus we believe that the inhibition of MAP kinases by chronic MKP3 expression must operate through a different mechanism.

In some cell types the phosphorylation of NET appears to trigger the internalization of NET (Jayanthi et al., 2004, 2006) and thus we tested the involvement of direct phosphorylation of NET in the phorbol ester and MKP3-mediated effects on trafficking. We find that removing the previously reported phosphorylation sites by mutagenesis does not prevent NET from being internalized in PC12 cells (Figure 1). This suggest that phosphorylation-dependent regulation of NET is cell type-dependent and not relevant to the type of regulation described in the present study. Other post-translational modifications such as ubiquitylation have recently been linked to the down regulation of DAT, because ubiquitylation of DAT occurs following activation of PKC and is required for its internalization (Miranda et al., 2005, 2007). On the other hand it has not been firmly established whether ubiquitylation also is involved in regulating NET activity. In our previous work we did not find any effect of MKP3 expression on the PMA-induced ubiquitylation of DAT (Mortensen et al., 2008) and in the present study we could not detect any basal or PKC-stimulated ubiquitinylation of NET. Thus, it appears that the mechanism responsible for down regulating transporter activity following PKC activation must differ between DAT and NET. This is perhaps not surprising as NET lacks all the N-terminal lysines that are ubiquitylated in DAT. We also conclude that ubiqutinylation of NET is not essential for MKP3-mediated effects on NET activity.

Using cell surface biotinylation assays and newly developed pharmacologic tools to assess endocytic mechanisms (Hill et al., 2009) we determined that PKC-activation leads to decreases in NET surface expression through a dynamin-dependent process (Figure 2). However, dynamin-dependence is not a consistent finding in all studies of NET and DAT internalization. As mentioned above the endocytosis of NET in placental trophoblasts involves direct phosphorylation of NET, however the process has been shown to be dynamin-independent (Jayanthi et al., 2004, 2006). In contrast several studies in cell lines as well as in dopaminergic neurons implicate dynamin in the regulation of surface expression of the DAT (Daniels and Amara, 1999; Sorkina et al., 2005; Gabriel et al., 2013). One report examining transfected DAT in PC12 cells noted that while PKC-mediated internalization appears to be dynamin-dependent, constitutive internalization can be dynamin-independent (Gabriel et al., 2013). Here, we demonstrate in PC12 cells that PKC-mediated decreases in NET surface expression proceed through a dynamin-regulated endocytic process that is inhibited by MKP3 expression (Figure 3).

The activity of MKPs has been shown to be highly specific for MAP kinases and they show very little activity to other serine/threonine phosphorylated proteins or tyrosine phosphorylated substrates (Alessi et al., 1993; Charles et al., 1993; Sun et al., 1993; Groom et al., 1996). A factor that contributes to this high specificity among the MKP family of phosphatases is the observation that the catalytic activity of MKP3 is increased dramatically when bound to its MAP kinase substrate (Camps et al., 1998). To our surprise, because the activation state of the kinases in PC12 cells does not correlate with the regulation of NET activity, we could rule out the direct involvement of all three-consensus MAP kinase families in the PKC-mediated regulation of NET and DAT (Figure 4). This result is similar to what we found for DAT regulation in both canine MDCK cells and Xenopus oocytes. We did find in PC12 cells that ERK is activated by PMA treatment. However, blocking this activation through the addition of a pharmacological inhibitor of the upstream MAP kinase kinase MEK1/2 had no effect on the PMA-induced down regulation of NET. In contrast the application of EGF which produces robust ERK activation through a non-PKC mediated pathway did not have any effect on NET activity in PC12 cells. Moreover, we did not detect any activation of the stress-activated MAP kinases by PMA treatment.

Because pharmacological inhibition of conventional MAP kinases did not alter PKC-mediated NET internalization, we next tested the hypothesis that MKP3 mediates its effect through the dephosphorylation of a substrate other than the major MAP kinase isoforms. To achieve this we employed a substrate trapping method to isolate MKP3 substrates. This method takes advantage of a mutated version of MKP3 in which the catalytic site cysteine has been mutated to a serine which eliminates phosphatase activity and dramatically enhances the interaction between the phosphatase and its substrates, an interaction that otherwise can be very transient and elusive (Flint et al., 1997; Blanchetot et al., 2005). We identified ERK1/2 as the predominant substrate of MKP3 and within the sensitivity of our assay we could not identify any other substrates. This suggests that the major isoforms ERK1/2 are the likely substrates of MKP3 and that these MAP kinases do not regulate transporter activity acutely, but instead, together with MKP3 they can regulate transporter activity through a chronic inhibition of ERK1/2 that lead to changes in transcription. We hypothesized that this permanent inhibition of ERK1/2 results in long term adaptive changes in PC12 cells that alter the mechanisms of PKC-mediated regulation of NET. We tested this hypothesis in two ways. First, we took advantage of a novel technology that enables the temporal control of active MKP3 protein in cells by introducing a destabilizing domain into MKP3 that increases its rate of degradation (Banaszynski et al., 2006; Egeler et al., 2011). In the presence of a small molecule named Shield1 the destabilized version of MKP3 is stabilized within hours. Using this system we showed that when MKP3 was acutely stabilized (for 4 h) it had no effect on PKC-mediated down regulation of NET activity. Second, we used a dominant negative mutant to inhibit Ras proteins, the upstream regulators of ERK1/2 signaling, as a means to inactivate ERK1/2 signaling for a longer duration. Expression of the dominant negative mutant RasN17 resulted in complete inhibition of PKC-activated down regulation of NET activity. Taken together these results suggest that MKP3 regulates NET activity by inhibiting MAP kinase signaling chronically, altering gene expression and making the NET resistant to PKC-mediated down regulation.

MAP kinases are essential for the transduction of extracellular signals to the nucleus of the cell and mediate many of their effects by regulating the expression of genes (Chen et al., 2001). To explore if changes in gene expression could be involved in the mechanism of MKP3-mediated NET regulation we used high-density DNA microarrays to establish the chronic effect of MKP3 on gene expression in PC12 cells. We found that the presence of MKP3 has a dramatic effect on overall gene expression, resulting in changes in the expression of several 100 genes. Most of the changes were in a negative direction with reduced expression in the presence of MKP3. These genes are strong candidates for genes that are regulated by ERK1/2. This is supported by the presence of several genes that are well-known to be regulated by ERK1/2 signaling (Lawrence et al., 2008). In some instances the changes were dramatic as some genes were turned completely off. To investigate whether any of these genes are responsible for MKP3-mediated regulation of NET activity we searched for regulated genes that were already implicated in membrane protein trafficking and discovered that caveolin-1 expression was completely silenced by MKP3 expression. Caveolins are important for some types of clathrin-independent endocytosis and are the major component of caveolae (Parton and Richards, 2003). This plasma membrane microdomain has already been shown to be involved in NET regulation in other cell types (Jayanthi et al., 2004). To test whether caveolins are involved in NET regulation in PC12 cells we used RNAi-based technologies to specifically knock down the expression of the caveolin-1 gene. This resulted in the regulation of NET activity that is comparable to what we found in the MKP3-expressing cells. Surprisingly, the effect of MKP3 expression was not rescued by re-introducing caveolin-1 expression in the MKP3 expressing PC12 cells. Taken together, this demonstrates a role for caveolins in regulating NET surface expression but does also suggest that other genes are involved in mediating the effects of MKP3 on PKC-mediated regulation of NET surface expression.

Because MKP3 and other MKPs are expressed at very low basal amounts in the brain but are turned on by activity (Boschert et al., 1998), we hypothesize that MKP3 expression physiologically function as an important regulator of neural plasticity including the membrane trafficking events that determine neurotransmitter transporter activity. As mentioned above experiments have established that intracellular NET can be recruited to the cell surface in states of high neuronal activity (Miner et al., 2006) and depolarization results in increased NET activity (Mandela and Ordway, 2006). This result implies that surface expression of NET is highly regulated. We believe the process we are reporting here mediated by MKP3 could be involved in this regulation. For example, it is known that MKP3 mRNAs are up regulated by acute and chronic treatment of rats with the NET substrate methamphetamine (Takaki et al., 2001). It could be speculated that the purpose of this up regulation is homeostatic to regulate the expression of genes that are involved in maintaining membrane proteins like NET on the surface of the neurons to enhance the termination of neurotransmitter signaling during periods of increased activity and in response to psychostimulant administration. We have indeed in another study found effects of MKP3 expression on a voltage gated calcium channel Cav1.2 that is also involved in neurotransmitter homeostasis (Mortensen, 2013). Finally, a recent study has shown that MKP1, another phosphatase that regulates ERK1/2 signaling, is up regulated in major depressive disorder (MDD) and that when MKP1 is absent in mice it enhances resilience to stress (Duric et al., 2010). As NET is a target of some of the most effective drugs employed in the treatment of depression, ADHD and chronic pain management it is tempting to speculate that disease associated alterations in ERK1/2-mediated signaling and the consequent changes in NET activity, such as those reported here, could have clinical significance.

## Author Contributions

OVM and MBL designed the study, performed experiments, and wrote the article. SGA designed the study and wrote the article. All authors analyzed the results and approved the final version of the manuscript.

## Conflict of Interest Statement

The authors declare that the research was conducted in the absence of any commercial or financial relationships that could be construed as a potential conflict of interest.
