# Supplementary figures and images for: MAP Kinase Phosphatase 3 (MKP3) Preserves Norepinephrine Transporter Activity by Modulating ERK1/2 Kinase-Mediated Gene Expression
Source: Front Cell Neurosci. 2017 Aug 22;11:253. doi: 10.3389/fncel.2017.00253 (PMC5572231; doi:10.3389/fncel.2017.00253)

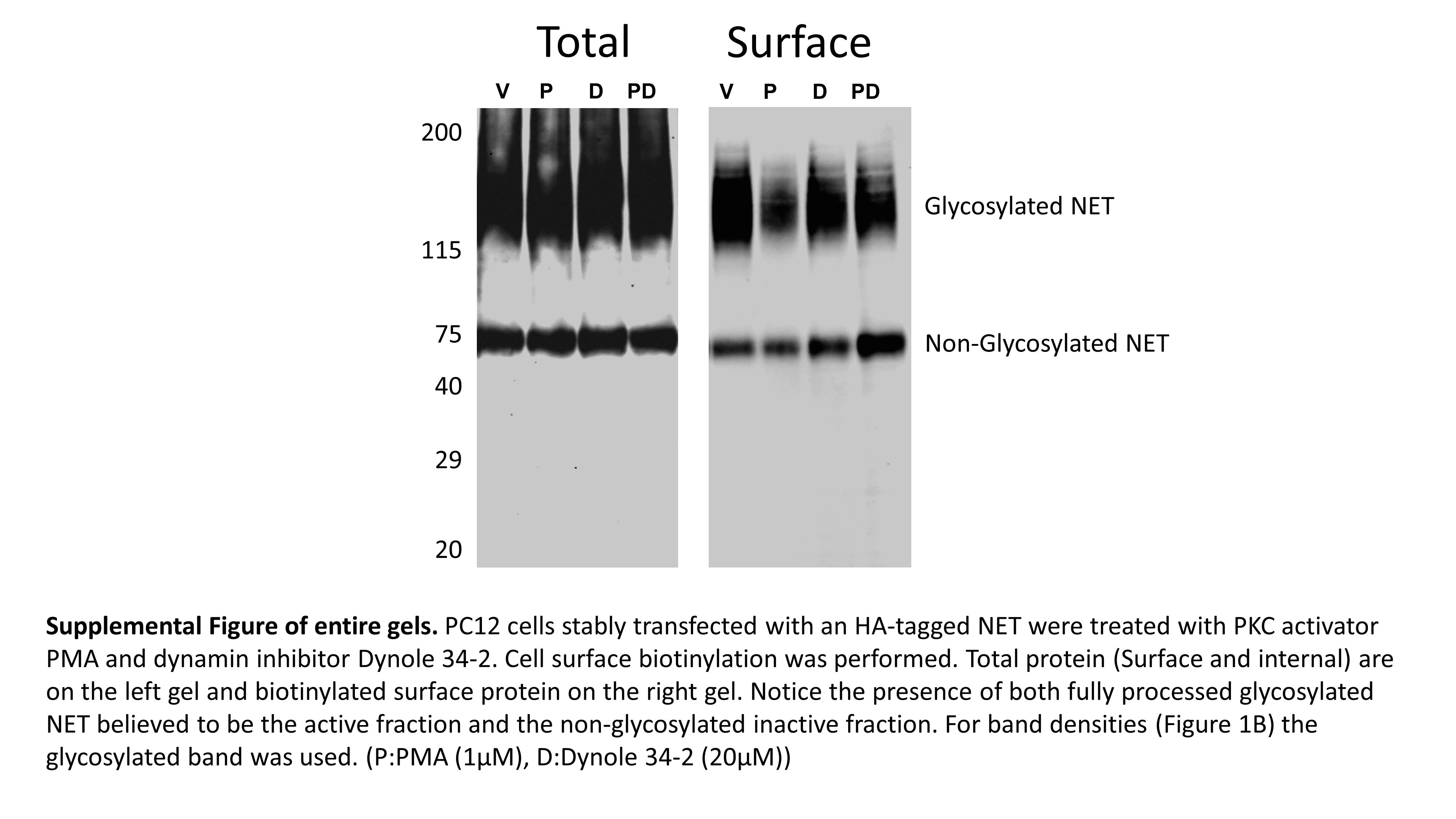

Supplement: Supplementary file 2 [file Image_1.tif]
